# Supplementary material for: The Use of Portion Control Plates to Promote Healthy Eating and Diet-Related Outcomes: A Scoping Review
Source: Nutrients. 2022 Feb 20;14(4):892. doi: 10.3390/nu14040892 (PMC8874720; doi:10.3390/nu14040892)
Supplement: Supplementary file 1 [file nutrients-14-00892-s001.zip › nutrients-1595282-supplementary.pdf]

Table S1: Search Strategy (Medline via Ovid)

	**Concept 1 intervention (portion control plate)**	**Concept 2 (diet-related outcomes)**
MeSH subject headings	Nil	Vegetables/<br>"diet, food, and nutrition"/<br>Diet/<br>Dietary Fiber/<br>Diet, Healthy/<br>Energy Intake/<br>Serving Size/<br>Portion Size/<br>Nutritional Requirements/<br>Feeding Behavior/<br>Nutritive Value/<br>Weight Loss/<br>Health Education/<br>exp Health Promotion/<br>Public Health/<br>Consumer Health Information/<br>Patient Education as Topic/<br>Health Behavior/
Keyword searches	(portion* adj2 (plate or plates)).tw.<br>(portion adj2 tool*).tw. (13)<br>((plate or plates) adj2 tool*).tw.<br>(Nestle portion plate* or My plate* or Eatwell plate*).tw.<br>((plate or plates) adj2 siz*).tw.<br>((food* or eat*) adj2 (plate or plates)).tw.<br>((plate or plates) adj1 model).tw.	behavio?r.tw.<br>((fruit* or vegetable* or heathy food*) adj2 (intake* or consum* or eat*)).tw.<br>((diet* or food*) adj2 guid*).tw.<br>(healthy adj2 eating).tw.<br>(healthy adj2 lifestyle).tw.<br>(guideline* adj2 adhere*).tw.<br>((eat* or diet* or intake) adj2 (pattern* or habit*)).tw.<br>diet* quality.tw.<br>portion size.tw.<br>portion control.tw.<br>(weight adj2 (loss or lose or reduction or reduc or maint* or control)).tw.<br>(promot* or educat* or intervention).tw.

Table S2: Quality Appraisal Table

First author, year	Question 1	Question 2	Question 3	Question 4	Question 5	Question 6	Question 7	Question 8	Question 9	Question 10	Question 11	Question 12	Question 13	Overall risk of bias[a]
Randomised controlled trials [b]														
Almiron-roig, 2016	Unclear	Unclear	Yes	No	No	Unclear	Yes	Unclear	Unclear	Yes	Yes	Yes	No	High
Bachman, 2013	No	Unclear	Unclear	No	Unclear	Unclear	Yes	Yes	Unclear	Yes	Yes	Yes	No	High
Bohnert, 2011	Unclear	Unclear	Yes	Yes	Unclear	Unclear	Yes	Yes	Yes	Yes	Yes	Yes	Yes	High
Ho, 2016	Yes	Yes	Yes	No	No	Unclear	Yes	Unclear	Yes	Yes	Yes	Yes	Yes	High
Huber, 2015	Yes	Yes	No	Yes	Yes	Yes	Yes	Yes	Yes	Yes	Yes	Yes	Yes	Low
Hughes, 2017 Study 1	Unclear	Unclear	Unclear	No	No	Unclear	Yes	Yes	Unclear	Yes	Yes	Yes	No	High
Hughes, 2017 Study 2	Unclear	Unclear	Unclear	No	No	Unclear	Yes	Yes	Unclear	Yes	Yes	Yes	No	High
Kesman, 2011	Yes	Yes	Yes	Yes	Unclear	Unclear	Yes	Yes	Yes	Yes	Yes	Yes	Yes	Low
Kline 2007	Unclear	Yes	Yes	Yes	Unclear	Unclear	Yes	Yes	Yes	Yes	Yes	Yes	Yes	High
Cross-sectional studies [c]														
Arcan, 2019	Yes	Unclear	Unclear	Yes	Yes	Unclear	Yes	Yes	N/A	N/A	N/A	N/A	N/A	High
Lara, 2015	Yes	Yes	Unclear	Unclear	Yes	Yes	Yes	Yes	N/A	N/A	N/A	N/A	N/A	Low
Nydahl, 1993	Yes	No	Unclear	Yes	No	No	Unclear	Unclear	N/A	N/A	N/A	N/A	N/A	High
Tagtow, 2017	Yes	No	No	Unclear	No	No	Unclear	Unclear	N/A	N/A	N/A	N/A	N/A	High
Wansink 2013	Yes	Yes	Unclear	Yes	Yes	No	Unclear	Yes	N/A	N/A	N/A	N/A	N/A	High
Quasi-experimental studies [d]														
Amaro, 2017	Yes	Yes	Yes	No	Yes	Yes	Yes	Unclear	Yes	N/A	N/A	N/A	N/A	Low
Blondin, 2018	Yes	Yes	Yes	No	Yes	Yes	Yes	Yes	Unclear	N/A	N/A	N/A	N/A	Low
Brown, 2014	Yes	Unclear	Yes	Yes	Yes	Unclear	Yes	Unclear	Yes	N/A	N/A	N/A	N/A	High
Edens, 2013	Yes	Unclear	Unclear	No	Yes	Yes	Yes	Yes	Yes	N/A	N/A	N/A	N/A	High
Ellsworth, 2014	Yes	Yes	Yes	No	Yes	No	Yes	Unclear	Yes	N/A	N/A	N/A	N/A	High
Melnick, 2018	Yes	Yes	Yes	No	Yes	No	Yes	Unclear	Yes	N/A	N/A	N/A	N/A	High
Shukaitis, 2021	Yes	Yes	Yes	Yes	No	Yes	Yes	Unclear	Yes	N/A	N/A	N/A	N/A	Low
Qualitative [e]														
Almiron-Roig, 2019	Unclear	Yes	No	Yes	Yes	No	No	Yes	Yes	Yes	Yes	N/A	N/A	High
Shilts 2015	Unclear	Yes	Unclear	No	Yes	No	No	Yes	Yes	Yes	N/A	N/A	N/A	High

[a] Studies were rated as high risk of bias if three or more criteria were Unclear or No, otherwise were rated as low risk of bias.

[b] The following 13 questions were used for randomised controlled trials. Question 1: Was true randomisation used for assignment of participants to treatment groups? Question 2: Was allocation to treatment groups concealed? Question 3: Were treatment groups similar at baseline? Question 4: Were participants blind to treatment assignment? Question 5: Were those delivering treatment blind to treatment assignment? Question 6: Were outcomes assessors blind to

treatment assignment? Question 7: Were treatment groups treated identically other than the intervention of interest? Question 8: Was follow up complete and if not, were differences between groups in terms of their follow up adequately described and analysed? Question 9: Were participants analysed in the groups to which they were randomised? Question 10: Were outcomes measured in the same way for treatment groups? Question 11: Were outcomes measured in a reliable way? Question 12: Was appropriate statistical analysis used? Question 13: Was the trial design appropriate, and any deviations from the standard RCT design (individual randomisation, parallel groups) accounted for in the conduct and analysis of the trial?

[c] The following 8 questions were used for cross-sectional studies. Question 1: Were the criteria for inclusion in the sample clearly defined? Question 2: Were the study subjects and the setting described in detail? Question 3: Was the exposure measured in a valid and reliable way? Question 4: Were objective, standard criteria used for measurement of the condition? Question 5: Were confounding factors identified? Question 6: Were strategies to deal with confounding factors stated? Question 7: Were the outcomes measured in a valid and reliable way? Question 8: Was appropriate statistical analysis used?
[d] The following 9 questions were used for quasi-experimental studies. Question 1: Is it clear in the study what is the 'cause' and what is the 'effect' (i.e. there is no confusion about which variable comes first)? Question 2: Were the participants included in any comparisons similar?

Question 3: Were the participants included in any comparisons receiving similar treatment/care, other than the exposure or intervention of interest? Question 4: Was there a control group? Question 5: Were there multiple measurements of the outcome both pre and post the intervention/ exposure? Question 6: Was follow up complete and if not, were differences between groups in terms of their follow up adequately described and analysed? Question 7: Were the outcomes of participants included in any comparisons measured in the same way? Question 8: Were outcomes measured in a reliable way? Question 9: Was appropriate statistical analysis used?

[e] The following 10 questions were used for qualitative studies. Question 1: Is there congruity between the stated philosophical perspective and the research methodology? Question 2: Is there congruity between the research methodology and the research question or objectives? Question 3: Is there congruity between the research methodology and the methods used to collect data? Question 4: Is there congruity between the research methodology and the representation and analysis of data? Question 5: Is there congruity between the research methodology and the interpretation of results? Question 6: Is there a statement locating the researcher culturally or theoretically? Question 7: Is the influence of the researcher on the research, and vice- versa, addressed? Question 8: Are participants, and their voices, adequately represented? Question 9: Is the research ethical according to current criteria or, for recent studies, and is there evidence of ethical approval by an appropriate body? Question 10: Do the conclusions drawn in the research report flow from the analysis, or interpretation, of the data?
